# Movement Strategies During the Penultimate and Final Steps of a Change of Direction Following Arthroscopic Partial Meniscectomy

**DOI:** 10.1111/sms.70367

**Published:** 2026-09-04

**Authors:** Chelsea Starbuck, Lee Herrington, Vanessa Walters, Bilal Barkatali, Richard Jones

**Affiliations:** ^1^ Applied Sports, Technology, Exercise and Medicine Research Centre, Faculty of Science and Engineering Swansea University Wales UK; ^2^ Human Movement and Rehabilitation, School of Health and Society University of Salford Salford UK; ^3^ The Manchester Institute of Health and Performance Manchester UK; ^4^ Blatchford Basingstoke UK; ^5^ The Orthteam Centre Spire Manchester Hospital Manchester UK

**Keywords:** limb sequence, meniscus injury, movement strategies, penultimate foot contact

## Abstract

Persistent knee offloading is well‐documented after arthroscopic partial meniscectomy (APM). Yet, it remains unclear if this extends to higher‐demanding change‐of‐direction (CoD) tasks and how step‐specific strategies influence joint loading. We aimed to compare joint power and relative contributions in the surgical limb, contralateral limb, and healthy individuals during a 90° CoD. A secondary aim examined the relationship between knee negative power at the penultimate and final foot contacts across limb sequences. Kinematic and kinetic data were collected from 24 post‐APM and 20 healthy individuals using motion capture with force plates. Participants performed a running approach before executing a 90° CoD. Linear mixed‐effects models compared limb differences and examined the relationship between steps. During penultimate contact, APM limb showed lower magnitudes of total (mean difference 2.80 W/kg, 95% CI 0.59, 5.00) and knee (2.60 W/kg, 0.75, 4.46) negative power than healthy limbs, with lower knee (−10.57%, −19.63, −1.52) and higher ankle (10.13%, 0.47, 19.79) contributions. During final contact, APM limb showed lower magnitudes of knee negative power (0.65 W/kg, 0.06, 1.24) than contralateral limbs. Compared with healthy, APM limb showed lower positive knee and ankle power, with lower knee (−7.23%, −14.19, −0.27) and greater hip (9.73%, 0.33, 19.13) contributions. Persistent knee deficits occurred post‐APM, characterized by ankle‐dominant braking and hip‐dominant propulsion. Limb sequence influenced knee loading, with APM penultimate contact shifting braking to the contralateral limb at final contact. These findings suggest rehabilitation approaches may benefit from prioritizing knee function while preparing the ankle and hip to tolerate redistributed loads.

## 1 Introduction

Traumatic meniscus injuries often occur in sports during high‐load movements such as rapid decelerations and changes of direction [1]. When the injury results in mechanical locking or occurs in avascular regions, arthroscopic partial meniscectomy (APM) is commonly performed to remove the damaged tissue [2, 3]. Although APM can facilitate a relatively quick return to sport [4], many individuals experience long‐term pain and dysfunction [5], and a greater risk of developing knee osteoarthritis [6].

Despite clearance to return to sport, individuals following APM often demonstrate a knee offloading strategy. Although meniscus removal is associated with increased local joint stress, studies have consistently reported lower sagittal knee joint moments during walking, running, and bilateral landing [7, 8, 9]. The post‐APM deficits in quadriceps strength, muscle atrophy, impaired neuromuscular control, and psychological barriers likely drive this offloading strategy [7, 10, 11, 12, 13]. Willy et al. [9] further observed a redistribution of the support moment away from the knee toward the hip during treadmill running. Similar proximal shifts have also been reported following anterior cruciate ligament reconstruction (ACLR) [14, 15]. Whether individuals following APM adopt comparable redistribution strategies, particularly during more demanding sport‐specific tasks such as change of direction (CoD), remains unclear.

Upon return to sport following an APM, individuals are required to repeatedly perform higher‐demanding tasks such as CoD. Sharper CoD (≥ 90°) places high mechanical demands on the lower limb, with faster performance linked to greater knee joint loading [16, 17, 18]. Given their tendency to offload the knee in lower‐demanding tasks, it is uncertain whether individuals post‐APM can tolerate these higher demands. Evidence from ACLR reconstruction indicates that athletes adopt a knee offloading strategy during 90° CoD [19], but whether a similar adaptation occurs and how these individuals compensate after APM remains unknown.

Changes of direction are multi‐step tasks, with braking demands distributed across successive foot contacts [20]. The penultimate foot contact (PFC) is integral, dissipating momentum and lowering the center of mass to prepare for effective redirection during the final foot contact (FFC) [20, 21, 22]. The PFC is characterized by greater posterior braking forces and sagittal knee joint loading than the FFC, contributing to larger reductions in velocity before redirection [20, 23]. Yet, despite its central role, the PFC has received little attention in post‐knee injury research, which has predominantly focused on the FFC. Insufficient PFC braking may shift greater eccentric demands onto the FFC, leading to diminished performance and increased risk of injury. For individuals following APM, persistent quadriceps weakness and knee offloading strategies may limit tolerance to these high braking loads across both PFC and FFC.

Given previous evidence, individuals following APM adopt a knee offloading strategy during lower‐demanding tasks, however, whether this strategy extends to higher‐demanding tasks, such as 90° CoD remains unknown. Examining lower limb joint power and the relative joint contributions provides insight into their movement strategies and how individuals redistribute load when knee demands are high. It is unclear whether offloading also occurs during the PFC. If such compensations arise, they may carry over into the contralateral limb during FFC, altering redirection mechanics, constraining performance, and increasing the risk of injury. The primary aim of this study was to compare joint power and relative contributions at the hip, knee, and ankle between the APM limb, the contralateral limb, and a healthy group during 90° CoD. Our secondary aim was to evaluate whether the relationship between knee joint negative power in the penultimate and final steps differs according to limb sequence (i.e., whether the APM or contralateral limb initiates the sequence).

## 2 Methods

Individuals who had undergone an arthroscopic partial meniscectomy (APM) and healthy individuals volunteered for this cross‐sectional study. All participants were required to be aged between 18 and 40 years, to participate in sport at least twice a week, and to be able to perform sport‐specific movements such as CoD. Participants were excluded if they had a history of inflammatory or infectious pathology in the lower limb or lower limb ligament laxity. Individuals who had undergone an APM were recruited post‐surgery through NHS and private clinics, with all procedures performed by experienced sport‐specific orthopedic surgeons. Participants were assessed 3–12 months following their surgery and included if they sustained an isolated traumatic meniscal injury during a sporting movement (e.g., change of direction, landing, or running). Participants who had undergone either a medial or lateral APM were eligible. In the APM group, participants were excluded if they had a history of lower extremity surgery (not including APM), prior lower limb traumatic injury unrelated to the sustained meniscal injury, or any history of contralateral lower limb injury or surgery. Healthy individuals were included if they had no history of lower limb surgery or traumatic lower limb injury. Ethical approval was obtained from the UK NHS Regional Ethical Committee (REC reference 18/EE/0015, IRAS: 239135). Informed consent was obtained from each participant before data collection.

To quantify kinesiophobia and knee function, participants completed the Tampa Scale of Kinesiophobia (TSK) [24] and the Knee injury and Osteoarthritis Outcome Score [25], further outlined by Starbuck et al. [7] Isometric quadriceps strength data were also assessed, with the participant seated with 85° hip flexion and 60° knee flexion on the isokinetic dynamometer (Biodex System 3 PRO; Biodex Medical Systems). Following four submaximal warm‐up familiarization repetitions, five maximum efforts held for 5 s with a 20‐s rest between repetitions were recorded. Maximum isometric torque was normalized to body mass [7].

**FIGURE 1:**
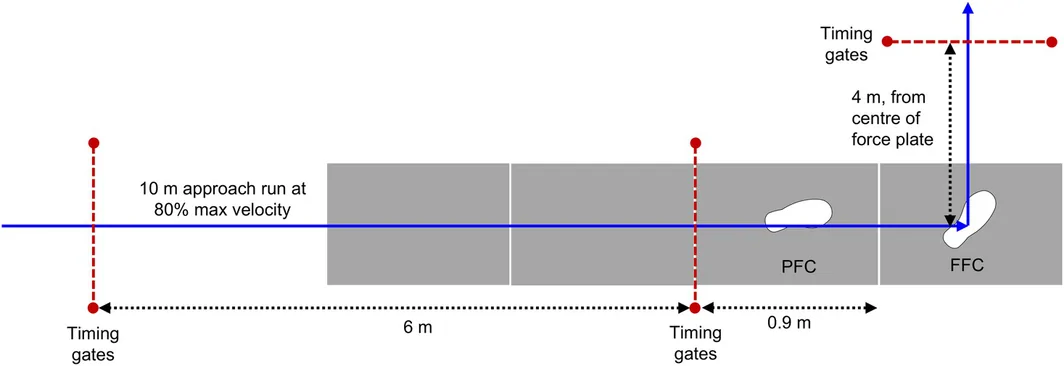
Schematic of the change of direction task highlighting the penultimate (PFC) and final (FFC) foot contacts. Blue arrow represents the direction of travel. Red dots and dashes represent timing gates. Gray shaded boxes represent force platforms.

Synchronized kinematic (200 Hz, 27 Qualisys Oqus, Qualisys, Gothenburg, Sweden) and kinetic (1000 Hz, AMTI force platforms, Advanced Mechanical Technology Inc., Newton, MA, USA) data were collected during a 90‐degree change of direction. Participants performed one maximal running effort prior to the CoD task to determine maximal running speed. Participants were then instructed to run 10 m at 80% of their maximal running speed before planting their foot on the force plate to perform a 90° CoD followed by running maximally 4 m through a set of cones (Figure 1). Timing gates (Witty, Microgate, Bolzano, Italy) were positioned 6 m apart prior to the CoD maneuver and 4 m from the center of the final force plate to monitor approach velocity. Participants were asked to perform three successful trials for both the left and right sides. A trial was deemed successful if there was clean foot contact on the force platform for the FFC and participants performed a straight run‐up without evidence of stuttering or mis‐stepping before the CoD FFC. Participants wore standardized footwear (Asics Gel‐Windhawk) during the assessment. Retroreflective markers were placed on anatomical landmarks on the participants' thorax and lower limbs bilaterally [7]. Rigid clusters with four non‐orthogonal markers were attached to the thighs and shanks bilaterally. A static calibration trial was collected before dynamic trials were recorded.

An inverse kinematic model was created in Visual3D (v6, C‐Motion, Germantown, MD, USA) [26]. The pelvis was assigned six degrees of freedom relative to the global coordinate system, while the thorax was assigned three rotational degrees of freedom relative to the pelvis. At the hip, knee, and ankle, translation constraints were applied. Weighted least squares global optimisation using the Levenberg–Marquardt algorithm was performed within Visual3D [26, 27]. A weight factor of four was applied to the thorax, pelvis, and foot, whilst the shank and thigh were set with a weight factor of three and two, respectively.

Butterworth low‐pass (4th order) filters with 15 Hz matched cutoff frequencies were applied to the marker and analogue data. A regression model estimated the hip joint centres based on anterior superior iliac spinae markers [28]. Hip, knee, and ankle kinematics were calculated from XYZ Carden angles. Geometric and inertial segment properties were estimated for each participant [29, 30]. Internal joint moments were initially calculated using inverse dynamics resolved to the proximal segment. Owing to the previously reported good to excellent reliability of sagittal plane kinematics and kinetics during 90° CoD [31], and evidence that the sagittal plane plays a larger role in joint power absorption and generation than other planes in this task [32], sagittal joint power time series were analyzed. Hip, knee, and ankle joint powers were calculated as the product of internal joint moment and joint angular velocity and normalized to body mass.

Average mechanical joint power and relative joint power contributions were calculated during the ground contact phases of PFC and FFC. The PFC refers to the second to last ground contact before the change of direction (i.e., contact immediately preceding FFC), and the FFC refers to the last contact before movement into the new intended direction. Ground contact was determined when the vertical ground reaction force exceeded 20 N. The negative and positive periods were analyzed separately as previously outlined [33] using a custom‐written MATLAB code (R2024a, MathWorks, Natick, Massachusetts, USA). Using the trapezoidal rule, individual joint powers were integrated with respect to time. Total joint work was calculated by summing all periods of positive and negative work separately. Total joint work was then divided by ground contact time to calculate average mechanical joint power. The total average lower limb power was calculated as the sum of the mechanical joint powers at the hip, knee, and ankle. Relative joint contributions were expressed as a percentage of total average lower limb power. For each outcome, the mean of three successful trials per limb was used for analysis.

To assess CoD performance between limbs, ground contact time for both steps was calculated. Centre of mass (COM) was determined from the trunk and lower limb model [34]. Horizontal COM velocity was obtained at initial contact and toe‐off for both steps to indicate approach and exit velocities [16, 34]. Approach and exit CoD angles were calculated from the horizontal COM displacements at initial contact and toe‐off for both steps [16, 35].

Due to missing data owing to poor force platform foot contact identified during data processing, the formal analysis included 24 APM limbs, 23 contralateral limbs, and 39 healthy limbs for the FFC, and 20 APM limbs, 20 contralateral limbs, and 35 healthy limbs for the PFC. Linear‐mixed effects models were used to compare between limbs, with participants included as random effects and limbs as fixed effects. Post hoc comparisons with 95% confidence intervals were performed using Holm's adjustments for multiple comparisons. A linear mixed‐effects model was used to examine the relationship between average negative knee mechanical power during the PFC and FFC, while accounting for limb sequence and within‐participant dependencies. PFC and FFC pairs were constructed based on limb sequences (i.e., APM to contralateral, contralateral to APM). The average negative mechanical knee power for FFC was the dependent variable. Fixed effects included average negative knee mechanical power during the PFC, limb sequence, and their interaction. To account for repeated measures within participants, a random intercept for participant ID was included. Statistical analyses were conducted in R (R Core Team 2025, v4.5.0) within RStudio (2025.05.1, RStudio, PBC, Boston, MA, USA) using the lme4, lmerTest, and emmeans packages [36, 37, 38].

## 3 Results

**FIGURE 2:**
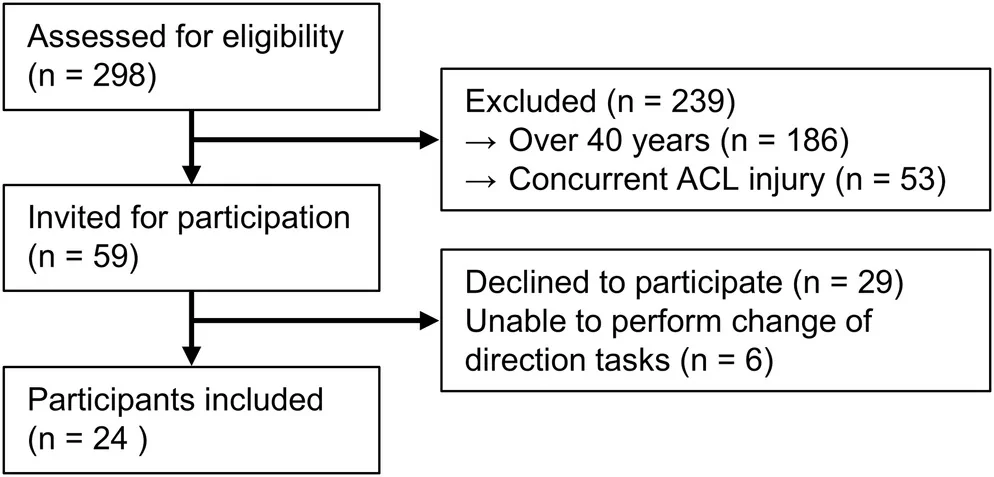
Flowchart of arthroscopic partial meniscectomy group. ACL, anterior cruciate ligament.

Twenty‐four individuals who had undergone an APM and 20 healthy individuals were assessed in the study (Figure 2). Participant characteristics and self‐reported outcomes are presented in Table 1. Differences were observed between groups for participant age, kinesiophobia, and perceived knee function. Additionally, the healthy group (3.3 ± 1.0 Nm/kg) demonstrated stronger isometric quadriceps strength compared to both APM limb (2.2 ± 0.7 Nm/kg; mean difference −1.0 Nm/kg, 95% CI −1.7, −0.3 Nm/kg, *p* = 0.002) and contralateral limbs (2.3 ± 0.8 Nm/kg; mean difference −0.9 Nm/kg, 95% CI −1.6, −0.2 Nm/kg, *p* = 0.003). No differences in isometric quadriceps strength were observed between APM and contralateral limbs (mean difference −0.1 Nm/kg, 95% CI −0.3, 0.1 Nm/kg, *p* = 0.286).

**TABLE 1:** Participant characteristics and self‐reported outcomes.

	Healthy group	APM group	Mean differences^a^ (95% CI)
Male/Female (*n*)	12/8	16/8	
Age (years)	24.3 (5.3)	30.2 (6.4)	5.9 (2.4, 9.5)
Height (m)	1.76 (0.08)	1.76 (0.10)	0.0 (−0.1, 0.1)
Mass (kg)	75.1 (10.8)	82.0 (12.9)	6.9 (−0.3, 14.1)
Medial/lateral/both medial & lateral APM (*n*)	N/A	10/12/2	
Time of assessment post APM (months)	N/A	6.0 (3.1)	
TSK	30.4 (4.9)	35.2 (6.7)	**4.7 (1.2, 8.2)**
KOOS	98.0 (2.3)	68.1 (18.0)	**−29.9 (−37.8, −22.1)**
Pain	99.0 (2.1)	74.6 (16.7)	**−24.4 (−31.7, −17.1)**
Symptoms	94.6 (5.1)	66.0 (19.9)	**−28.7 (−37.5, −19.8)**
Activities of daily living	99.9 (0.5)	85.7 (14.2)	**−14.2 (−20.3, −8.0)**
Sport and recreation	97.8 (5.7)	62.4 (24.5)	**−35.4 (−46.2, −24.5)**
Quality of life	98.8 (3.3)	51.6 (24.4)	**−47.1 (−57.8, −36.5)**

*Note:* Bold values indicate significant difference between groups compared (*p* < 0.05).

Abbreviations: APM, arthroscopic partial meniscectomy; CI, confidence interval; KOOS, Knee injury and Osteoarthritis Outcome score; TSK, Tampa Scale of Kinesiophobia.

^a^ Mean differences reported as APM group – Healthy group.

### 3.1 Change of Direction Outcomes

No differences for CoD performance outcomes were observed across foot contacts except for PFC ground contact time (Table 2). The contralateral limb had longer ground contact times compared to the healthy group (*p* = 0.026) and the APM limb (*p* = 0.010).

**TABLE 2:** Mean and SD for change of direction performance outcomes for penultimate and final foot contact across groups.

	Healthy	Meniscectomy		Mean differences (95% CI)		
		APM	Contralateral	APM—healthy	APM—contralateral	Contralateral—healthy
Penultimate foot contact						
Approach velocity (m/s)	3.86 (0.44)	3.49 (0.65)	3.62 (0.63)	−0.36 (−0.78, 0.06)	−0.13 (−0.30, 0.05)	−0.23 (−0.64, 0.19)
Exit velocity (m/s)	2.96 (0.36)	2.78 (0.61)	2.69 (0.52)	−0.18 (−0.54, 0.18)	0.06 (−0.10, 0.23)	−0.24 (−0.61, 0.12)
Ground contact time (s)	0.22 (0.04)	0.23 (0.04)	0.26 (0.04)	0.00 (−0.02, 0.03)	**−0.03 (−0.05, −0.01)**	**0.03 (0.00, 0.06)**
Approach CoD angle (°)	4.09 (2.02)	5.10 (2.98)	3.50 (2.03)	1.00 (−0.66, 2.66)	1.57 (−0.05, 3.18)	−0.57 (−2.23, 1.09)
Exit CoD angle (°)	11.33 (5.29)	11.97 (4.75)	11.20 (4.64)	0.49 (−3.22, 4.20)	0.58 (−2.26, 3.41)	−0.08 (−3.79, 3.63)
Final foot contact						
Approach velocity (m/s)	2.92 (0.37)	2.77 (0.46)	2.78 (0.57)	−0.15 (−0.48, 0.19)	−0.02 (−0.15, 0.10)	−0.12 (−0.46, 0.21)
Exit velocity (m/s)	2.71 (0.43)	2.54 (0.37)	2.56 (0.41)	−0.17 (−0.46, 0.12)	−0.04 (−0.22, 0.14)	−0.13 (−0.43, 0.16)
Ground contact time (s)	0.34 (0.05)	0.34 (0.08)	0.33 (0.07)	0.00 (−0.05, 0.05)	0.01 (−0.01, 0.03)	−0.01 (−0.05, 0.04)
Approach CoD angle (°)	9.92 (5.67)	10.41 (4.42)	9.93 (5.10)	0.48 (−3.21, 4.19)	0.36 (−2.01, 2.73)	0.12 (−3.59, 3.83)
Exit CoD angle (°)	61.68 (6.80)	59.31 (10.05)	60.65 (9.25)	−2.33 (−8.46, 3.81)	−0.95 (−4.29, 2.40)	−1.38 (−7.55, 4.79)

*Note:* Bold values indicate significant difference between groups compared (*p* < 0.05).

Abbreviations: APM, arthroscopic partial meniscectomy; CI, confidence interval; CoD, change of direction.

### 3.2 Penultimate Foot Contact

**FIGURE 3:**
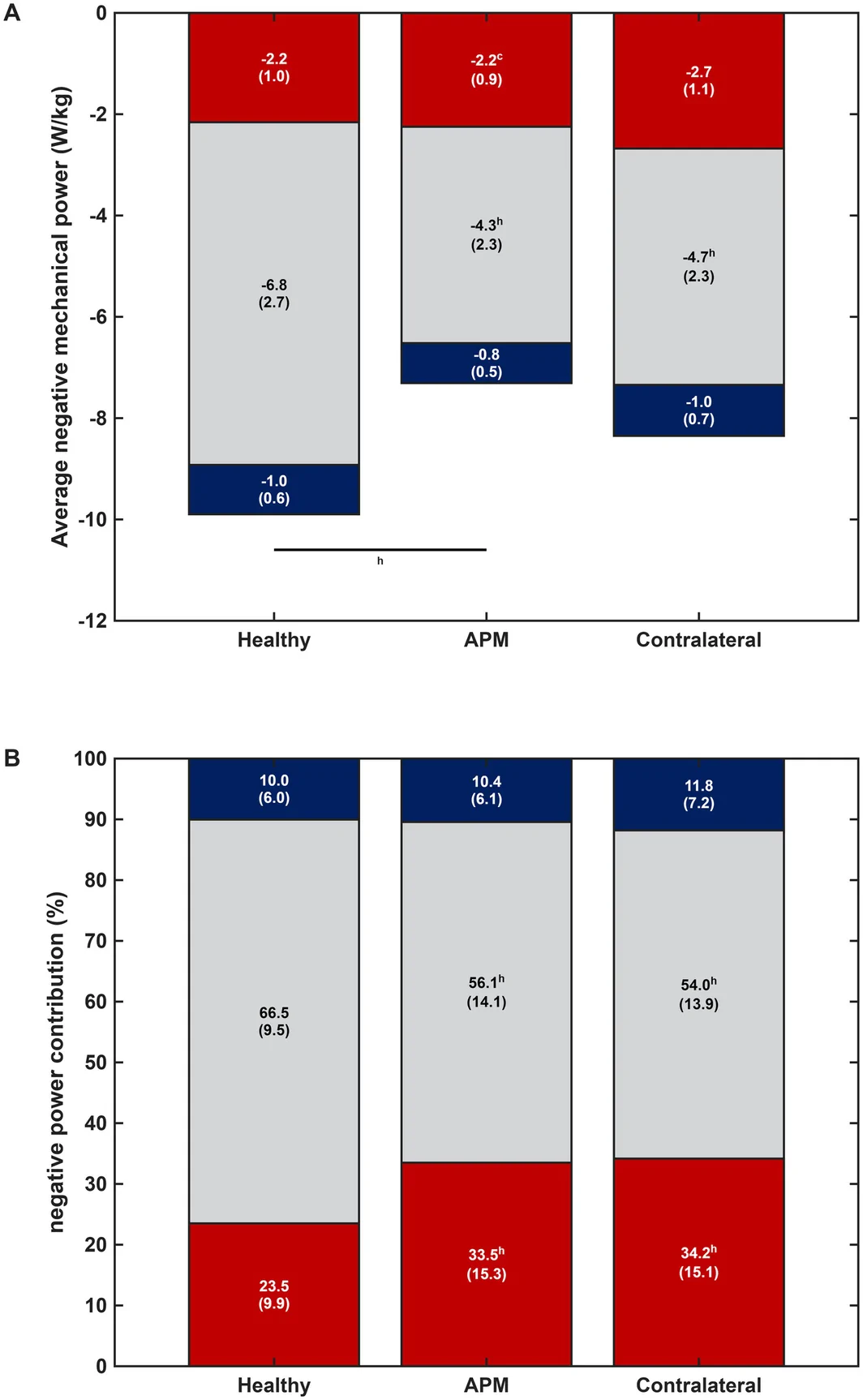
Mean and standard deviation (SD) for negative mechanical power for each limb (healthy, APM [arthroscopic partial meniscectomy], and contralateral) at the ankle (red), knee (gray), and hip (blue) joints. (A) Presents absolute average mechanical powers. (B) Presents relative joint contributions (%). ^*h*^Significantly different (*p* < 0.05) from the healthy group, and ^*c*^significantly different (*p* < 0.05) from the contralateral leg.

During the PFC, total negative average mechanical power was lower in the APM limb compared to the healthy group (*p* = 0.007; Figure 3A), detailed further in the Supporting Information (Tables S1 and S2). Lower negative average mechanical knee power was observed in the APM (*p* = 0.003) and contralateral limbs (*p* = 0.022) compared to the healthy group. Average negative mechanical power for the ankle was lower in the APM limb compared to the contralateral limb (*p* = 0.036). Relative negative knee joint contributions were lower in the APM limb (*p* = 0.015) and contralateral limbs (*p* = 0.006) compared to the healthy group (Figure 3B). Additionally, relative negative ankle joint contributions were higher in the APM limbs (*p* = 0.032) and contralateral limbs (*p* = 0.028) compared to the healthy group.

### 3.3 Final Foot Contact

**FIGURE 4:**
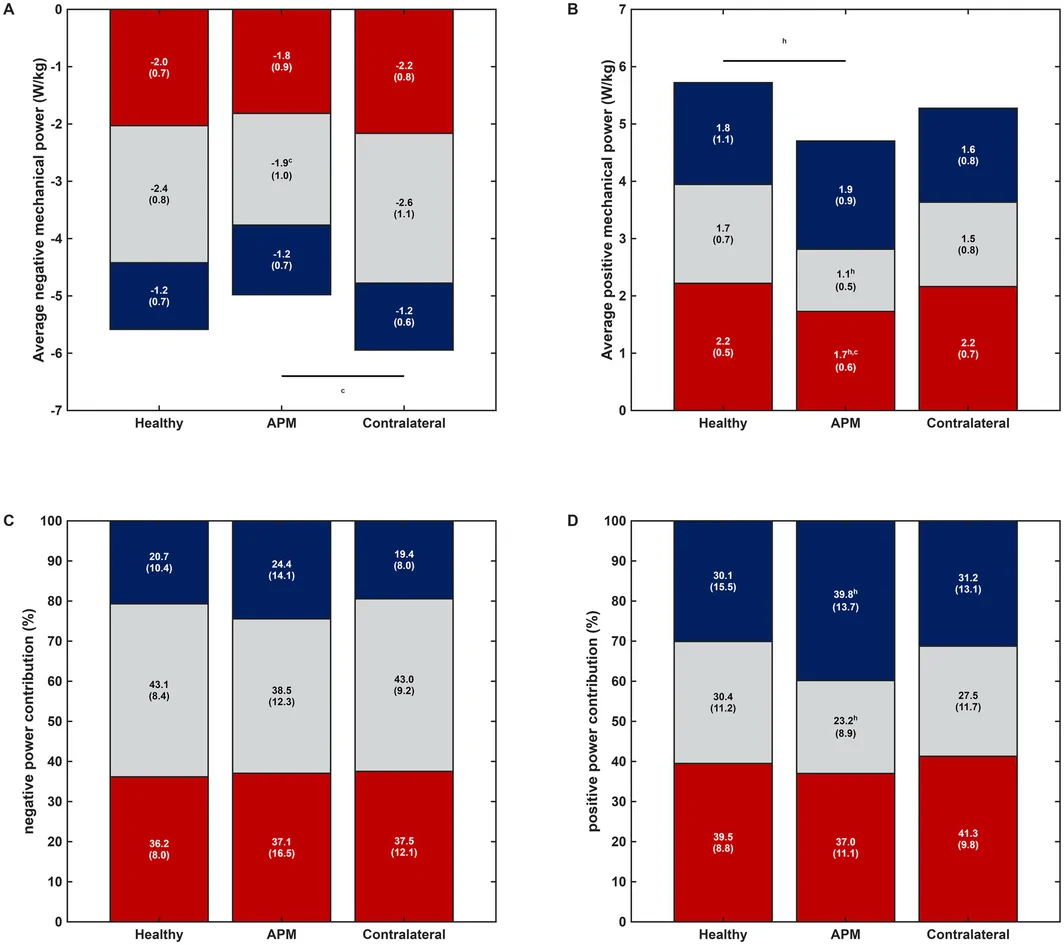
Mean and standard deviation (SD) for negative (A, C) and positive (B, D) mechanical power for each limb (healthy, APM [arthroscopic partial meniscectomy], and contralateral) at the ankle (red), knee (gray), and hip (blue) joints. (A, B) Presents absolute average mechanical powers. (C, D) Presents relative joint contributions (%). ^*h*^Significantly different (*p* < 0.05) from the healthy group, and ^*c*^significantly different (*p* < 0.05) from the contralateral leg.

During the FFC, total average negative mechanical power was lower in the APM limb compared to the contralateral limb (*p* = 0.031; Figure 4A). Lower negative average mechanical knee power was observed in the APM (*p* = 0.023) compared to the contralateral leg. No differences in limbs were observed for the relative negative joint contributions during the FFC (Figure 4C). Total average positive mechanical power was lower in the APM limb compared to the healthy group (*p* = 0.008; Figure 4B). Lower positive average mechanical knee power was observed in the APM (*p* = 0.004) compared to the healthy group. Positive average mechanical ankle power was lower in the APM limbs compared to the healthy group (*p* = 0.018) and contralateral limbs (*p* = 0.004). Relative positive knee joint contributions were lower in the APM limb (*p* = 0.034) compared to the healthy group (Figure 4D). Additionally, relative positive hip joint contributions were higher in the APM limbs (*p* = 0.035) compared to the healthy group.

### 3.4 Limb Sequence Across Steps Interaction

**FIGURE 5:**
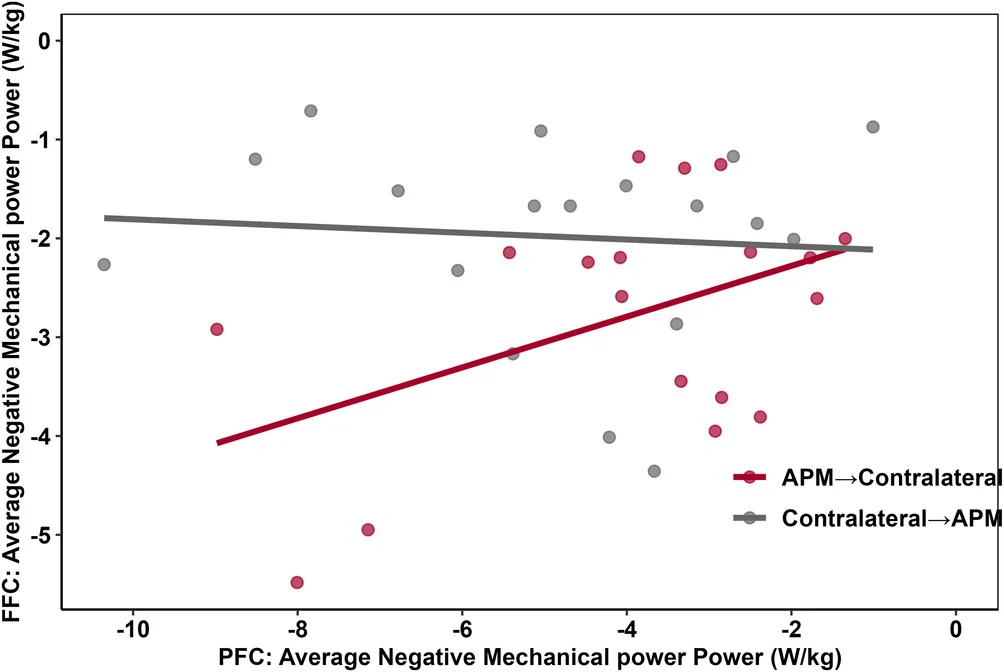
Relationship between average negative mechanical power for penultimate foot contact (PFC) and final foot contact (FFC) during a change of direction. Each point represents paired steps for APM (arthroscopic partial meniscectomy) to contralateral (red), and contralateral to APM (gray).

The model examining the relationship between average negative mechanical knee power across PFC and FFC (Figure 5) revealed a significant interaction with limb sequence (*β* = −0.35, SE 0.11, *p* = 0.008). When the APM preceded the contralateral limb, the association between PFC and FFC power was positive but non‐significant (*β* = 0.16, SE 0.11, 95% CI −0.07, 0.39, *p* = 0.149). When the contralateral limb preceded the APM limb, the association was negative but also non‐significant (*β* = −0.19, SE 0.10, 95% CI −0.39, 0.02, *p* = 0.07). Pairwise comparison of these slopes confirmed a significant difference between limb sequence (mean difference = 0.35, SE = 0.11, 95% CI 0.11, 0.59, *p* = 0.007).

## 4 Discussion

Individuals who have undergone an APM demonstrated lower capacity to dissipate and generate energy in their surgical limb across both the penultimate and final step of a 90° CoD, compared to their contralateral limb and a healthy group. These deficits were characterized by lower average mechanical power during braking and propulsion, driven by lower knee power, and the adoption of an altered movement strategy. Knee contribution shifted distally during braking in the PFC and proximally during propulsion in the FFC. The relationship between average negative mechanical knee power in the PFC and FFC differed depending on limb sequence (i.e., APM to contralateral or contralateral to APM), indicating that eccentric knee control across steps is influenced by which limb initiates the change of direction sequence, a factor not previously examined in knee injury research.

Our findings show that, post‐APM, both the surgical and contralateral limbs exhibited lower knee power during the PFC compared to the healthy limb, consistent with known bilateral quadriceps weakness previously reported [7] and reported within this study. The bilateral deficits may reduce both limbs' capacity to tolerate high eccentric braking loads during the PFC [20], potentially increasing risk of muscle atrophy without affecting overall CoD performance. Such bilateral deficits contrast with previous APM studies, which predominantly reported APM‐limb specific knee offloading only [7, 8, 9]. These studies assessed lower‐demanding tasks (e.g., walking, running, bilateral landing) that may not impose sufficient braking demands to reveal contralateral deficits [7, 8, 9]. The present findings suggest that bilateral deficits may become apparent during higher‐demanding braking, such as during the PFC of a 90° CoD, but may remain undetected during lower‐demanding assessments. Therefore, incorporating higher‐demand braking tasks during return to sports assessments could better evaluate limb‐specific braking capacity.

Joint power contributions during the PFC indicated an altered movement strategy in both the APM and contralateral limbs compared with the healthy limb, characterized by a distal redistribution from the knee to the ankle during this high‐braking phase. This redistribution reflects a compensatory braking strategy in response to reduced eccentric knee capacity. Greater negative ankle power and longer ground contact time during the PFC in the contralateral limb compared to the APM limb suggest that the contralateral limb may utilize a longer braking to increase distal energy absorption. In contrast, the higher relative ankle contribution in the APM limb likely reflects reduced knee involvement rather than greater ankle power. While ankle‐dominant braking could mitigate knee loading, these compensations could increase eccentric demand on the ankle joint and influence the braking demands during the FFC. Therefore, rehabilitation strategies should consider targeting bilateral eccentric knee and ankle capacity.

Our secondary analysis revealed a significant limb sequence interaction in the relationship between PFC and FFC knee negative mechanical power. When the contralateral limb led, greater knee braking in the PFC was associated with less APM knee braking in the FFC, consistent with evidence highlighting the penultimate step's role in offloading the knee and enabling an effective, safer FFC [20]. However, when the APM led, greater PFC braking was associated with greater contralateral FFC braking. In the PFC, bilateral quadriceps weakness may limit braking capacity in both limbs; however, the offloading of the APM limb likely reflects limb‐specific compensatory strategies, with factors such as kinesiophobia potentially contributing. Higher loading in FFC has been associated with increased knee injury risk [18], suggesting compensatory patterns in PFC expose the contralateral limb during the FFC. Rehabilitation strategies should consider the role of the PFC and the capacity for the APM limb to load sufficiently to minimize risk of contralateral limb injury.

During the FFC propulsion phase, the APM limb produced lower total positive limb power than the healthy limb, driven by reduced knee and ankle power. This was accompanied by a redistribution of work away from the knee and toward the hip, with a greater proportion of hip contribution compared to healthy. This proximal shift was similar to that reported during the propulsion phase of a double‐leg drop jump after ACLR [15], where diminished quadriceps function led to greater reliance on the hip extensors. While greater hip involvement has been associated with improved performance during 45° CoD [16, 17], in our cohort, this strategy did not fully restore propulsion capacity, as total limb power remained lower in the APM limb. Reliance on the hip may protect the knee in the short term, but if unaddressed, could perpetuate quadriceps weakness and atrophy, ultimately limiting future return to sport performances.

This is the first study to evaluate penultimate and final foot contacts during a higher‐demanding CoD following APM. Certain limitations should be considered. We examined a pre‐planned 90° CoD to ensure controlled procedures and participant safety. Yet, in sport, unanticipated maneuvers occur and are known to alter braking strategies across steps [39], warranting future studies. Change of direction angle also influences lower limb loading and performance outcomes [40]. A 90° CoD was selected as it is considered highly demanding on the knee [17], providing new insights into individuals' ability to tolerate these loads and the compensatory strategies they adopt post‐APM. Nevertheless, examining a wider CoD range of angles would improve ecological validity. Participants were assessed between 3 and 12 months post‐surgery and met the eligibility criteria, including the ability to perform sport‐specific tasks such as CoD; however, variability in recovery status may influence movement strategies. Owing to resource constraints, our sample size was limited. A sensitivity analysis indicated that within‐participant comparisons and between‐participant comparisons were sufficiently powered to detect effect sizes of at least 0.66 and 0.86, respectively. Joint power and relative power contributions offer valuable insight into inter‐joint and inter‐limb compensations across penultimate and final steps; these analyses represent net joint estimates rather than individual muscle contributions. While this limits the interpretation of specific muscle strategies, joint powers are valuable in identifying movement adaptations following APM. The use of the contralateral limb as a comparator is often considered a limitation due to potential bilateral adaptations following injury. Our findings support this, showing that the contralateral limb also demonstrated altered knee loading compared to healthy individuals. This reinforces the need for future studies to include both contralateral and healthy comparators when examining post‐APM movement strategies.

## 5 Conclusions

Following an APM, individuals demonstrated persistent knee power deficits across both the penultimate and final foot contacts of a 90° change of direction. These deficits resulted in altered, step‐dependent movement strategies. During the penultimate step, individuals adopted a distal ankle strategy in both APM and contralateral limbs. During the final step, the APM limb relied on a proximal hip strategy, likely to aid propulsion. Limb sequence influenced knee braking demands; when the APM led, greater PFC braking was associated with greater contralateral FFC braking. This suggests a compensatory strategy to shift demand away from the APM limb toward the contralateral limb, which could increase the risk of injury in the contralateral limb. Rehabilitation strategies that restore knee power while developing ankle and hip capacity to tolerate high joint loads of these demanding movements should be considered and examined further in future studies.

## 6 Perspective

Longitudinal research is needed to determine how lower‐limb loading strategies following APM develop over time and how they relate to quadriceps strength, symptoms, and function, particularly during higher‐demanding tasks. Further work should examine whether current approaches to return to sport assessment adequately evaluate an individual's capacity to tolerate higher sporting demands and which outcomes may better inform return to sport criteria following APM. Intervention‐based research is also needed to determine whether rehabilitation focused on restoring knee loading capacity and quadriceps strength while preparing the whole lower limb to tolerate higher sporting demands can improve function. Future studies should carefully consider the use of the contralateral limb as a comparator where bilateral adaptations may be present. Assessment of CoD should also consider loading across both limbs and the CoD sequence, including the penultimate step, rather than focusing only on the operated limb or final foot contact. Examining these movement strategies during unanticipated maneuvers and under fatigue would better reflect the demands experienced during sport.

## Author Contributions

**Chelsea Starbuck:** conceptualisation, data curation, formal analysis, investigation, methodology, visualization, writing – original draft. **Lee Herrington:** conceptualisation, visualization, writing – original draft. **Vanessa Walters:** investigation, data curation, writing – review and editing. **Bilal Barkatali:** resources, writing – review and editing. **Richard Jones:** conceptualisation, supervision, writing – review and editing.

## Funding

The authors have nothing to report.

## Ethics Statement

REC reference 18/EE/0015, IRAS: 239135.

## Conflicts of Interest

The authors declare no conflicts of interest.

## Supporting information


**Table S1:** Mean and SD for change of direction percentage power contributions for penultimate and final foot contact across groups.
**Table S2:** Mean and SD for change of direction average mechanical powers for penultimate and final foot contact across groups.

## Data Availability

The data that support the findings of this study are available from the corresponding author upon reasonable request.
