# Supplementary material for: Movement Strategies During the Penultimate and Final Steps of a Change of Direction Following Arthroscopic Partial Meniscectomy
Source: Scand J Med Sci Sports. 2026 Sep 4;36(9):e70367. doi: 10.1111/sms.70367 (PMC13543057; doi:10.1111/sms.70367)
Supplement: Supplementary file 1 [file SMS-36-e70367-s001.docx]

# Supplementary Material (S1)

Table S1: Mean and SD for change of direction percentage power contributions for penultimate and final foot contact across groups

|  |  | **Meniscectomy** | |  | ***Mean differences (95% CI)*** | | |
| --- | --- | --- | --- | --- | --- | --- | --- |
|  | **Healthy** | **APM** | **Contralateral** |  | ***APM - Healthy*** | ***APM - Contralateral*** | ***Contralateral - Healthy*** |
| *Penultimate foot contact* |  |  |  |  |  |  |  |
| Negative hip power (%) | 10.04 (5.96) | 10.43 (6.10) | 11.82 (7.22) |  | 0.45  (-4.38, 5.28) | -1.02  (-4.00, 1.96) | 1.47  (-3.36, 6.30) |
| Negative knee power (%) | 66.45 (9.52) | 56.06 (14.15) | 54.01 (13.89) |  | **-10.57**  **(-19.63, -1.52)** | 1.27  (-5.05, 7.59) | **-11.84**  **(-20.89, -2.78)** |
| Negative ankle power (%) | 23.51 (9.88) | 33.50 (15.32) | 34.17 (15.09) |  | **10.13**  **(0.47, 19.79)** | -0.20  (-7.00, 6.59) | **10.33**  **(0.67, 19.99)** |
| *Final foot contact* |  |  |  |  |  |  |  |
| Negative hip power (%) | 20.69 (10.36) | 24.44 (14.12) | 19.44 (8.01) |  | 3.76  (-3.71, 11.23) | 4.99  (-1.47, 11.45) | -1.23  (-8.78, 6.32) |
| Negative knee power (%) | 43.14 (8.36) | 38.51 (12.32) | 43.03 (9.23) |  | -4.67  (-11.20, 1.87) | -4.52  (-10.77, 1.74) | -0.15  (-6.76, 6.46) |
| Negative ankle power (%) | 36.16 (7.98) | 37.05 (16.54) | 37.53 (12.13) |  | 0.95  (-7.54, 9.43) | -0.48  (-6.00, 5.04) | 1.43  (-7.12, 9.97) |
| Positive hip power (%) | 30.08 (15.49) | 39.83 (13.66) | 31.25 (13.08) |  | **9.73**  **(0.33, 19.13)** | 8.59  (-1.08, 18.25) | 1.14  (-8.38, 10.66) |
| Positive knee power (%) | 30.42 (11.17) | 23.17 (8.91) | 27.46 (11.66) |  | **-7.23**  **(-14.19, -0.27)** | -4.30  (-11.68, 3.09) | -2.94  (-9.98, 4.11) |
| Positive ankle power (%) | 39.50 (8.76) | 37.00 (11.13) | 41.29 (9.81) |  | -2.54  (-9.43, 4.35) | -4.29  (-8.79, 0.21) | 1.75  (-5.18, 8.68) |

**APM, arthroscopic partial meniscectomy, CI, confidence interval, bold values indicate significant difference between groups compared (*P* < .05).**

Table S2. Mean and SD for change of direction average mechanical powers for penultimate and final foot contact across groups

|  |  | **Meniscectomy** | |  | ***Mean differences (95% CI)*** | | |
| --- | --- | --- | --- | --- | --- | --- | --- |
|  | **Healthy** | **APM** | **Contralateral** |  | ***APM - Healthy*** | ***APM - Contralateral*** | ***Contralateral - Healthy*** |
| *Penultimate foot contact* |  |  |  |  |  |  |  |
| Negative hip power (W/kg) | -0.97 (0.57) | -0.79 (0.54) | -1.01 (0.71) |  | 0.22  (-0.24, 0.67) | 0.21  (-0.05, 0.47) | 0.00  (-0.45, 0.46) |
| Negative knee power (W/kg) | -6.76 (2.75) | -4.27 (2.34) | -4.67 (2.32) |  | **2.60**  **(0.75, 4.46)** | 0.55  (-1.09, 2.18) | **2.06**  **(0.20, 3.91)** |
| Negative ankle power (W/kg) | -2.16 (0.99) | -2.25 (0.93) | -2.68 (1.10) |  | -0.04  (-0.80, 0.71) | **0.49**  **(0.01, 0.96)** | -0.53  (-1.28, 0.22) |
| Total negative power (W/kg) | -9.91 (3.27) | -7.31 (2.67) | -8.35 (2.68) |  | **2.80**  **(0.59, 5.00)** | 1.28  (-0.47, 3.03) | 1.52  (-0.69, 3.72) |
| *Final foot contact* |  |  |  |  |  |  |  |
| Negative hip power (W/kg) | -1.16 (0.65) | -1.21 (0.74) | -1.17 (0.56) |  | -0.06  (-0.52, 0.40) | -0.06  (-0.39, 0.27) | 0.00  (-0.46, 0.47) |
| Negative knee power (W/kg) | -2.39 (0.78) | -1.95 (0.95) | -2.61 (1.14) |  | 0.44  (-0.18, 1.06) | **0.65**  **(0.06, 1.24)** | -0.21  (-0.84, 0.42) |
| Negative ankle power (W/kg) | -2.03 (0.75) | -1.82 (0.92) | -2.16 (0.79) |  | 0.20  (-0.36, 0.77) | 0.33  (-0.10, 0.76) | -0.12  (-0.69, 0.45) |
| Total negative power (W/kg) | -5.58 (1.48) | -4.98 (1.34) | -5.94 (1.78) |  | 0.59  (-0.47, 1.64) | **0.91**  **(0.05, 1.78)** | -0.33  (-1.39, 0.74) |
| Positive hip power (W/kg) | 1.78 (1.10) | 1.89 (0.86) | 1.64 (0.82) |  | 0.11  (-0.52, 0.75) | 0.25  (-0.40, 0.90) | -0.14  (-0.78, 0.50) |
| Positive knee power (W/kg) | 1.73 (0.69) | 1.09 (0.50) | 1.47 (0.82) |  | **-0.63**  **(-1.09, -0.17)** | -0.38  (-0.80, 0.03) | -0.25  (-0.72, 0.22) |
| Positive ankle power (W/kg) | 2.22 (0.52) | 1.73 (0.65) | 2.16 (0.70) |  | **-0.48**  **(-0.91, -0.06)** | **-0.43**  **(-0.74, -0.11)** | -0.06  (-0.48, 0.37) |
| Total positive power (W/kg) | 5.72 (1.15) | 4.70 (1.10) | 5.27 (1.14) |  | **-1.00**  **(-1.78, -0.21)** | -0.55  (-1.17, 0.07) | -0.44  (-1.24, 0.35) |

**APM, arthroscopic partial meniscectomy, CI, confidence interval, bold values indicate significant difference between groups compared (*P* < .05).**
